# Downstream clinical consequences of stress cardiovascular magnetic resonance based on appropriate use criteria

**DOI:** 10.1186/s12968-015-0137-x

**Published:** 2015-05-15

**Authors:** Sloane McGraw, Omer Mirza, Michael A Bauml, Vibhav S Rangarajan, Afshin Farzaneh-Far

**Affiliations:** Section of Cardiology, Department of Medicine, University of Illinois at Chicago, 840 South Wood St. M/C 715, Suite 920 S, Chicago, IL 60612 USA; Division of Cardiology, Department of Medicine, Duke University Medical Center, Durham, NC USA

## Abstract

**Background:**

Appropriate use criteria (AUC) have been developed by professional organizations as a response to the rising costs of imaging, with the goal of optimizing test-patient selection. Consequently, the AUC are now increasingly used by third-party-payers to assess reimbursement. However, these criteria were created by expert consensus and have not been systematically assessed for CMR. The aim of this study was to determine the rates of abnormal stress-CMR and subsequent downstream utilization of angiography and revascularization procedures based on the most recent AUC.

**Methods:**

300 consecutive patients referred for CMR-stress testing were prospectively enrolled. Two cardiologists reviewed all clinical information before the CMR-stress test and classified the test as “appropriate’, “maybe appropriate” or “rarely appropriate” according to the 2013 AUC. Patients were followed for 2 months for the primary outcomes of coronary angiography and/or revascularization.

**Results:**

49.7% of stress CMRs were appropriate, 36.7% maybe appropriate, and 13.6% rarely appropriate. Ischemia was significantly more likely to be seen in the appropriate (18.8%) or maybe appropriate groups (21.8%) than the rarely appropriate group (4.8%) (*p* = 0.030 and *p* = 0.014 respectively). Referral for cardiac catheterization was not significantly different in the appropriate (10.1%) and maybe appropriate groups (10.0%) compared to the rarely appropriate group (2.4%) (*p* = 0.119 and *p* = 0.127 respectively). No patients undergoing catheterization in the rarely appropriate group went on to require revascularization, in contrast to 53.3% of the appropriate vs 36.4% of the maybe appropriate patients (*p* = 0.391). Presence of ischemia led to referral for cardiac catheterization in 50.0% of the appropriate group vs 33.3% of the maybe appropriate group (*p* = 0.225); in contrast to none of the rarely appropriate group.

**Conclusions:**

The great majority of tests were classified as appropriate or maybe appropriate. Downstream cardiac catheterization rates were similar in all 3 groups. However, rarely appropriate studies never required revascularization, suggesting suboptimal resource utilization. Studies classified as maybe appropriate had similar rates of abnormal findings and led to similar rates of downstream catheterization and revascularization as those that were deemed appropriate. This suggests that consideration could be given to upgrading some of the common maybe appropriate indications to the appropriate category.

## Background

As a response to fiscal pressures and with the goal of optimizing test-patient selection, appropriate use criteria (AUC) have been developed by several professional organizations - including the American College of Cardiology, the Society for Cardiovascular Magnetic Resonance, the American Society of Nuclear Cardiology and the American Society of Echocardiography [1–5]. Consequently, the AUC are now increasingly used by payers to assess the suitability of reimbursement for imaging procedures. Moreover, recent approval of the “Protecting Access to Medicare Act” (PAMA) by the United States Congress has very significant implications for the application of AUC to CMR reimbursement [6,7]. It states that starting in 2017 - in order to be paid by Medicare for advanced imaging - certification must be provided that the ordering physicians consulted the AUC. The claim to Medicare must specify whether the requested imaging adheres to the AUC. The act also states that criteria must be developed by national medical societies or provider-led entities and should be evidence based, to the extent possible. By November 2015, the Centers for Medicare and Medicaid Services (CMS) must select the specific AUC to be used. Finally, in 2020 CMS must identify up to 5% of physicians as outliers in adherence to AUC, who may then be subject to prior authorization.

Stress CMR is increasing used in the management of patients with known or suspected coronary artery disease [8]. The initial AUC for stress CMR was recently replaced by the AUC for stable ischemic heart disease, as part of a new multimodality approach [1,5]. The AUC were developed by a panel who were asked to rate 80 clinical indications as being “appropriate”, “maybe appropriate”, or “rarely appropriate”. However, these criteria were created by expert consensus and have never been systematically assessed or validated for CMR. In particular, there is no data regarding the impact of the AUC on downstream cardiac procedures.

The aims of this study were to determine the rates of abnormal stress CMR results and subsequent downstream utilization of angiography and revascularization procedures based on the most recent AUC.

## Methods

### Study population

Three hundred consecutive patients referred for CMR stress testing were prospectively enrolled in a single academic medical center. Patients were excluded if they had metallic implants incompatible with CMR, glomerular filtration rate < 30 ml/min, high degree atrio-ventricular block, severe active wheezing from asthma or severe claustrophobia. Subjects were asked to abstain from caffeine-containing products for at least 12 h prior to the test.

### Clinical variables

Demographic and clinical characteristics were recorded prospectively at the time of CMR stress testing from patient interviews and the electronic medical record. Patients gave informed written consent for the protocol, which was approved by the local institutional review board.

### CMR acquisition

Images were acquired on a 3 T scanner (Philips Achieva, Philips Medical Systems, Best, the Netherlands) using a six-element phased-array receiver coil as previously described [9]. Steady-state free-precession cine images were acquired in multiple short-axis and three long-axis views (repetition time, 3.0 ms; echo time, 1.5 ms; flip angle, 40°; slice thickness 6 mm).

The patient table was then partially pulled outside the scanner bore to allow direct observation of the patient and full access. A 0.4 mg bolus of regadenoson (Lexiscan, Astellas Pharma Inc) was infused under continuous electrocardiography and blood pressure monitoring. Approximately 1 min after regadenoson administration, the perfusion sequence was applied and Gadolinium contrast (0.075 mmol/kg gadoteridol, Bracco Diagnostics) followed by a saline flush (30 ml) was infused (4.5 ml/s) via an antecubital vein. On the console, the perfusion images were observed as they were acquired, with breath-holding starting from the appearance of contrast in the right ventricular cavity. Imaging was completed 10 to 15 s after the gadolinium bolus had transited the left ventricular myocardium. Perfusion images consisted of three to four short-axis slices obtained every heartbeat with a saturation-recovery, gradient-echo sequence (repetition time 2.8 ms; echo time 1.1 ms; flip angle 20°; voxel size, 2.5 × 2.5 × 8 mm). Aminophylline (100 mg IV) was administered immediately after stress perfusion imaging [9]. Rest perfusion images were acquired 15 min after stress imaging with an additional contrast bolus (0.075 mmol/kg gadoteridol) using identical sequence parameters. Five minutes after rest perfusion, late gadolinium enhancement (LGE) imaging was performed with a 2D segmented gradient echo phase-sensitive inversion-recovery sequence in the identical views as cine-CMR. Inversion delay times were typically 280 to 360 ms. Perfusion and LGE images were visually interpreted by standard methods [10]. The pattern of LGE was classified as either infarct or non-CAD type on the basis of subendocardial enhancement [11,12].

### Patient classification for AUC

Two independent general cardiologists reviewed all clinical information dated before the CMR stress test. These reviewers were blinded to the results of the CMR and to the clinical course subsequent to the test. The CMR stress tests were classified as “appropriate’, “maybe appropriate” or “rarely appropriate” as defined by the 2013 AUC [5]. A third blinded independent physician adjudicated any discrepancy between the interpreters.

### Follow-up

After CMR, patients were prospectively followed for 2 months for the primary outcomes of coronary angiography and/or revascularization. Clinical follow-up was based on review of the electronic medical records or from telephone interviews with patients or their physicians.

### Statistical analysis

Normally distributed data were expressed as mean ± SD. Continuous variables were compared by the Student’s *t*-test or Wilcoxon rank-sum (depending on data normality). Comparisons of discrete variables were made using the chi-square test; Fisher’s exact test was used when the assumptions of the chi-square test were not met. A p value of <0.05 was considered statistically significant.

## Results

### Patient characteristics

Table 1 summarizes the baseline patient characteristics. The mean age of the study population was 59 years. 54% of patients were female and 36% had diabetes mellitus. 32% had known coronary artery disease, including prior PCI (17%) and CABG (4%). The mean ejection fraction was 61%. Of the 300 CMRs reviewed, arbitration for AUC assignment by a third cardiologist was required for just 1 patient.

**Table 1:** Baseline Characteristics Stratified by Appropriate Use Criteria

CHARACTERISTICS	Total N = 300	Appropriate N = 149	Maybe Appropriate N = 110	Rarely Appropriate N = 41	P Value
**Age** (±SD)	59 (±13.6)	61 (±12.0)	61 (±13.3)	46 (±14.4)	<0.0001
**Female %**	54.0	49.7	55.0	66.7	0.1703
**BMI** (±SD)	30.8 (±5.7)	31.3 (±5.4)	30.8 (±6.1)	29.2 (±5.5)	0.1000
**Diabetes %**	36.2	43.6	32.4	16.7	0.0049
**Hyperlipidemia %**	53.6	60.4	55.9	23.8	0.0002
**Current Smoking %**	16.3	18.8	8.5	27.8	0.0089
**Hypertension %**	73.0	78.5	74.8	45.2	0.0002
**Known CAD %**	32.1	39.6	29.7	11.9	0.0032
**Prior MI %**	13.6	16.1	13.5	4.8	0.1793
**Prior PCI %**	16.9	22.8	14.4	2.4	0.0061
**Prior CABG %**	4.3	3.4	7.3	0	0.1058
**LVEF** (±SD)	61 (±10.9)	60.3 (±12.0)	61 (±10.7)	62 (±6.8)	0.4800

*BMI*, Body Mass Index; *CAD*, Coronary Artery Disease; *LVEF*, Left Ventricular Ejection Fraction; *MI*, myocardial infarction; *PCI*, Percutaneous Coronary Intervention; *CABG*, Coronary Artery Bypass Grafting; *SD*, standard deviation

### Primary outcomes

At 2 months of follow-up, the endpoint of coronary angiography occurred in 27 patients and the endpoint of revascularization occurred in 12 patients (PCI = 11, CABG = 1).

### Appropriateness

Based on the 2013 AUC, 49.7% of stress CMRs were classified as appropriate, 36.7% as maybe appropriate, and 13.6% as rarely appropriate. A comparison of the baseline characteristics across AUC is given in Table 1. There were significant differences in age, prevalence of diabetes, hyperlipidemia, smoking, hypertension, and known CAD across the three groups.

The six most frequent AUC categories accounted for 179 patients (Table 2). Of patients with these common six indications approximately 69% were classified as appropriate and 31% as maybe appropriate.

**Table 2:** AUC categories in our study population

AUC Description	N	Classification
Follow-up testing (>90 days) for new or worsening symptoms with non-obstructive CAD on coronary angiography (invasive or noninvasive) OR normal prior stress imaging study	48	A
Symptomatic in intermediate pre-test probability of CAD with interpretable ECG AND able to exercise	32	M
Symptomatic in intermediate pre-test probability of CAD with uninterpretable ECG OR unable to exercise	27	A
Newly diagnosed systolic heart failure (resting LV function previously assessed but no prior CAD evaluation)	25	A
Evaluation for symptomatic (ischemic equivalent) post-revascularization (PCI or CABG)	24	A
Sequential or follow up testing (≤90 days) with uncertain results on prior stress imaging study (not stress CMR) where obstructive CAD remains a concern	23	M
Symptomatic in low pre-test probability of CAD with interpretable ECG AND able to exercise	20	R
Pre-operative clearance in poor or unknown functional capacity (<4 METS); intermediate risk surgery with ≥1 clinical risk factor	12	M
High pre-test probability of CAD with an interpretable ECG and able to exercise	8	A
High pre-test probability of CAD with an uninterpretable ECG and unable to exercise	6	A
Follow up testing for new or worsening symptoms with an abnormal prior stress imaging study	6	M
Follow up testing (>90 Days) in an asymptomatic or symptomatically stable patient whose last study was ≥ 2 years ago	4	M
Follow up testing (>90 Days) in an asymptomatic patient without ischemic equivalent, who has a normal prior stress imaging study or non-obstructive CAD on angiogram who is intermediate to high global CAD risk with a study ≥ 2 years ago	4	M
Follow up testing (>90 Days) in an a patient with stable symptoms, who has a normal prior stress imaging study or non-obstructive CAD on angiogram who is intermediate to high global CAD risk with a study ≥ 2 years ago	4	M
Symptomatic patients who are low pre-test probability of CAD with an uninterpretable ECG or unable to exercise	3	M
Newly diagnosed diastolic heart failure	3	A
Evaluation of arrhythmias without ischemic equivalent with frequent PVCs	3	M
Syncope without ischemic equivalent in a patient with low global CAD risk	3	R
Follow up testing (>90 Days) in an asymptomatic or symptomatically stable patient with a history of abnormal prior stress imaging study < 2 years ago	3	R
Follow up testing (>90 Days) in an asymptomatic patient with a normal prior stress imaging study OR non-obstructive CAD on angiogram	3	R
Follow up testing for new or worsening symptoms in a patient with prior obstructive CAD on invasive coronary angiography	3	M
Pre-op risk stratification in a patient with poor or unknown functional capacity (<4 METs) in a patient who is undergoing vascular surgery with ≥ 1 clinical risk factor	3	M

*AUC*, Appropriate Use Criteria; *A*, Appropriate, *M*, Maybe Appropriate; *R*, Rarely Appropriate; *CAD*, Coronary Artery Disease; *ECG*, Electrocardiogram; *LV*, Left Ventricular; *PCI*, Percutaneous Coronary Intervention; *CABG*, Coronary Artery Bypass Grafting; *PVC*, Premature Ventricular Beat

### Relationship of appropriateness to CMR results

**Fig. 1:**
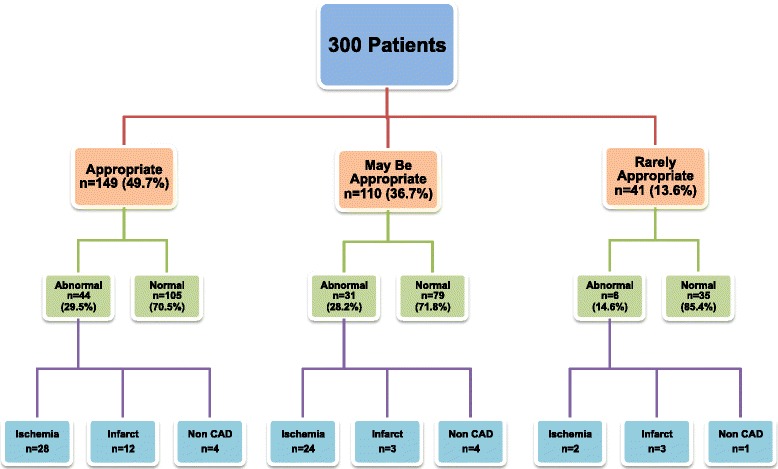
Appropriate use criteria and stress-CMR findings in the study population

**Fig. 2:**
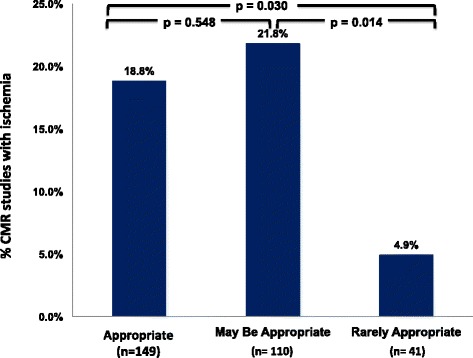
Studies with ischemia categorized by appropriate use criteria

Abnormal CMR stress results (defined as presence of ischemia or scar) were more common in the appropriate (29.5%) and maybe appropriate (28.2%) groups compared with the rarely appropriate (14.6%) group although the differences did not reach statistical significance (*p* = 0.055 and *p* = 0.085 respectively) (Fig. 1). Ischemia was significantly more likely to be seen in patients in the appropriate (18.8%) or maybe appropriate groups (21.8%) than the rarely appropriate group (4.8%) (*p* = 0.030 and *p* = 0.014 respectively) (Fig. 2).

### Relationship of appropriateness to downstream angiography and revascularization

**Fig. 3:**
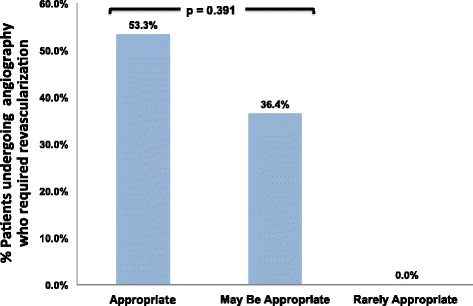
Patients undergoing cardiac catheterization who required revascularization, categorized by appropriate use criteria

There was a trend towards more referral for cardiac catheterization in the appropriate (10.1%) and maybe appropriate groups (10.0%) compared to the rarely appropriate group (2.4%), but the differences were not statistically significant (*p* = 0.119 and *p* = 0.127 respectively). However, none of the patients undergoing catheterization in the rarely appropriate group went on to require revascularization, in contrast to 53.3% of the appropriate vs 36.4% of the maybe appropriate patients (*p* = 0.391) (Fig. 3).

### Relationship of abnormal CMR results with referral to downstream angiography

The finding of ischemia on CMR led to referral for cardiac catheterization in 50.0% of the appropriate group vs 33.3% of the maybe appropriate group (*p* = 0.225). In contrast none of the rarely appropriate patients with ischemia were referred for cardiac catheterization. Based on an angiographic cut-off of ≤50% major epicardial stenosis, stress-CMR had a sensitivity = 94%, specificity = 44%, positive predictive value = 77%, and negative predictive value = 80%, in this biased referral population.

## Discussion

Due to rising healthcare costs, appropriate use of cardiovascular imaging is increasingly emphasized by professional societies, third party payers and accreditation agencies [6,7,13,14]. To the best of our knowledge this is the first study to systematically and prospectively assess the downstream utilization of angiography and revascularization procedures based on the AUC for stress-CMR. We have shown that tests categorized by the AUC as rarely appropriate, infrequently demonstrate ischemia, but the rates of downstream cardiac catheterization were not significantly different to those categorized as appropriate or maybe appropriate. Importantly when patients underwent cardiac catheterization none of the rarely appropriate group went on to require subsequent revascularization. These findings appear to support the current AUC classification of rarely appropriate stress-CMR studies, since this group appears to result in suboptimal resource utilization.

In our study population, the great majority of tests ordered were deemed as appropriate or maybe appropriate. Only, approximately 14% of the stress-CMRs were categorized as rarely appropriate. This is similar to the inappropriate proportions reported for stress echocardiography (9–33%) [15,16], Single Photon Emission Computed Tomography (SPECT) (7–46%) [17–19], and Positron Emission Tomography (PET) (10.2%) [20]. It is interesting to speculate whether growing pre-certification demands by third-party payers may have affected physicians test orderings patterns, but this cannot be assessed from this study.

We found that studies that were classified as maybe appropriate had similar rates of ischemia and led to similar rates of downstream catheterization and revascularization as those that were deemed appropriate. This suggests that consideration could be given to upgrading some of the common maybe appropriate indications to the appropriate category (Table 2).

It is interesting to note that even when ischemia was reported, patients were more likely to be referred for cardiac catheterization in the appropriate and maybe appropriate groups. In fact none of the patients with ischemia in the rarely appropriate group were referred for cardiac catheterization. The reasons for this are unclear but may relate to physician’s assessment that invasive testing and revascularization would not significantly change outcomes or symptoms in this patient group.

In this study we have looked at the rates of abnormal stress tests and the endpoints of downstream angiography and revascularization to help assess optimal test-patient selection and imaging utilization. However, there are a number of important caveats to bear in mind. Higher rates of abnormal findings and greater use of angiography or revascularization doesn’t necessarily imply better outcomes. Such validation would require performance of prospective randomized outcome trials. Ideally these studies should be part of larger initiatives to compare the effectiveness of different imaging modalities. Although, such studies will be challenging to perform and fund, they are of critical importance in clarifying optimal imaging strategies. Another important point to emphasize is that stress testing can be very useful in patient management even when it does not lead to angiography or revascularization [21]. For example a normal study may lead to exclusion of coronary artery disease as a cause of symptoms, as well as to clinic/hospital discharge; or it may lead to ‘surgical clearance’ in patients referred prior to elective non-cardiac surgery. Further studies are required to more comprehensively assess these types of clinical impact and management change [22,23].

Future steps in assessing and validating AUC for stress-CMR should aim to compare the prognostic ability of the test across the various AUC categories. Such an approach was recently undertaken by Doukky et al. in a large nuclear study [19]. They demonstrated that inappropriate use of SPECT was associated with reduced prognostic value. In those patients whose scans were appropriate or uncertain, abnormal scans were of significant value in predicting major adverse cardiac events (hazard ratio 3.1–3.7) compared with normal scans. However, in those with inappropriate scans, abnormal studies did not achieve significance in predicting adverse cardiac events. Moreover, all abnormal SPECT findings were associated with increasing rates of revascularization, irrespective of the level of appropriateness.

### Limitations

Our study was limited by a small sample size (*n* = 300) drawn from a single academic institution and may not be representative of the wider population. However, this may have the advantage of providing uniform scanning, interpretation and follow-up protocols. Larger studies with greater statistical power and more events (particularly revascularization) will allow a more comprehensive analysis of subgroups. The results of this study should therefore be regarded as preliminary, until larger multicenter studies are completed. As mentioned above, cardiac catheterization and revascularization are only part of the downstream clinical consequences of CMR-stress. Cost-effectiveness was not assessed in this study and clearly needs to be the subject of future studies aiming to establish the validity of the AUC.

## Conclusions

The great majority of tests ordered in our population were classified as appropriate or maybe appropriate. Downstream referral for cardiac catheterization was not significantly different in the 3 groups. However, rarely appropriate studies never required revascularization, suggesting suboptimal resource utilization. Studies classified as maybe appropriate had similar rates of abnormal findings and led to similar rates of downstream catheterization and revascularization as those that were deemed appropriate. This suggests that consideration could be given to upgrading some of the common maybe appropriate indications to the appropriate category.
